# Describing the Diapause-Preparatory Proteome of the Beetle *Colaphellus bowringi* and Identifying Candidates Affecting Lipid Accumulation Using Isobaric Tags for Mass Spectrometry-Based Proteome Quantification (iTRAQ)

**DOI:** 10.3389/fphys.2017.00251

**Published:** 2017-04-26

**Authors:** Qian-Qian Tan, Wen Liu, Fen Zhu, Chao-Liang Lei, Daniel A. Hahn, Xiao-Ping Wang

**Affiliations:** ^1^Hubei Insect Resources Utilization and Sustainable Pest Management Key Laboratory, College of Plant Science and Technology, Huazhong Agricultural UniversityWuhan, China; ^2^Department of Entomology and Nematology, University of FloridaGainesville, FL, USA

**Keywords:** diapause preparation, iTRAQ, proteomics, lipid metabolism, fatty acid-binding protein, stress resistance

## Abstract

Prior to entering diapause, insects must prepare themselves physiologically to withstand the stresses of arresting their development for a lengthy period. While studies describing the biochemical and cellular milieu of the maintenance phase of diapause are accumulating, few studies have taken an “omics” approach to describing molecular events during the diapause preparatory phase. We used isobaric tags and mass spectrometry (iTRAQ) to quantitatively compare the expression profiles of proteins identified during the onset of diapause preparation phase in the heads of adult female cabbage beetles, *Colaphellus bowringi*. A total of 3,175 proteins were identified, 297 of which were differentially expressed between diapause-destined and non-diapause-destined female adults and could therefore be involved in diapause preparation in this species. Comparison of identified proteins with protein function databases shows that many of these differentially expressed proteins enhanced in diapause destined beetles are involved in energy production and conversion, carbohydrate metabolism and transport, and lipid metabolism. Further hand annotation of differentially abundant peptides nominates several associated with stress hardiness, including HSPs and antioxidants, as well as neural development. In contrast, non-diapause destined beetles show substantial increases in cuticle proteins, suggesting additional post-emergence growth. Using RNA interference to silence a fatty acid-binding protein (FABP) that was highly abundant in the head of diapause-destined females prevented the accumulation of lipids in the fat body, a common product of diapause preparation in this species and others. Surprisingly, RNAi against the FABP also affected the transcript abundance of several heat shock proteins. These results suggest that the identified differentially expressed proteins that play vital roles in lipid metabolism may also contribute somehow to enhanced hardiness to environmental stress that is characteristic of diapause.

## Introduction

Diapause is a pre-programmed developmental arrest that allows insects to survive unfavorable environmental conditions and synchronize their growth and reproduction with favorable environmental conditions and abundant food resources (Tauber et al., 1986; Denlinger, 2002). Diapause can be divided into the following stages; pre-diapause (induction and preparation), diapause (initiation, maintenance, and termination) and post-diapause, each of which is characterized by distinct, dynamic physiological processes (Koštál, 2006). There is a considerable published literature on the molecular mechanisms associated with diapause (Denlinger, 2002; Zhang et al., 2012; Poelchau et al., 2013). However, most of this literature focuses on the diapause maintenance stage and the molecular events governing other stages of diapause in most insects remain poorly understood, including the diapause preparatory stage.

Insects undergo a series of physiological changes during the diapause preparatory phase, when they enter an alternative developmental trajectory that often results in increased nutrient accumulation and increased stress hardiness prior to arresting development in the diapause maintenance phase (Hahn and Denlinger, 2007, 2011; MacRae, 2010; Poelchau et al., 2013; Poupardin et al., 2015). For insects with a facultative diapause, environmental stimuli perceived during the diapause induction phase determine whether an individual will enter the diapause developmental trajectory or not (Sim and Denlinger, 2013; Omura et al., 2016). These environmental stimuli are translated into endocrine signals that regulate developmental arrest, nutrient accumulation, and stress resistance during the diapause preparation phase (Hahn and Denlinger, 2007; MacRae, 2010). However, exactly how these signals are transferred between the induction and preparation phases, and how they regulate diapause preparation remains unknown. Molecular processes that occur during diapause preparation, especially those involved in lipid metabolism, have recently attracted considerable research interest (Hahn and Denlinger, 2007, 2011; Denlinger and Armbruster, 2014; Sinclair, 2015). Although not uniformly true, diapause-destined individuals accumulate greater nutrient reserves than non-diapause individuals in many insect species. For example in adult *Culex pipiens* mosquitoes destined for reproductive diapause were found to accumulate higher lipid levels than their non-diapausing counterparts in the first week after adult emergence (Sim and Denlinger, 2009, 2013; Zhou and Miesfeld, 2009). It has been hypothesized that the decision of whether to enter diapause or not, as well as the decision of when an insect should remain in diapause or exit diapause and resume development may be affected by their nutrient stores (Tauber et al., 1986; Saunders, 1997; Menu and Desouhant, 2002; Xu et al., 2012), but the degree to which nutrient accumulation during the diapause preparatory phase affects diapause induction has yet to be well interrogated at the molecular level.

Previous studies of molecular mechanisms of diapause regulation have largely focused on the transcriptional level (Ragland et al., 2010, 2011; Poelchau et al., 2013; Qi et al., 2015; Kučerová et al., 2016; Meyers et al., 2016), with relatively few studies at the protein level (Zhang et al., 2012, 2013). Although proteins are the primary functional molecules in many different physiological processes, there is relatively little information on the differential expression of proteins across the phases of diapause. Changes in protein expression during diapause have mainly been explored using 2-dimensional gel electrophoresis (2-DE). A total of no more than 100 diapause-related proteins have been detected using 2-DE gel-based comparative proteomic and phosphoproteomic analyses in the flesh fly *Sarcophaga crassipalpis* (Pavlides et al., 2011), the corn borer *Sesamia nonagrioides* (Pérez-Hedo et al., 2012), and the cotton bollworm *Helicoverpa armigera* (Zhang et al., 2012, 2013). A new method, isobaric tags for relative and absolute quantitation (iTRAQ), has been developed that can quantitatively analyze proteins from various sources in a single assay with high sensitivity using tandem mass spectrometry (Ross et al., 2004). One clear difference between iTRAQ and 2-DE gel-based proteomics is that iTRAQ typically generates relative abundance data for many more proteins in each sample. For example, proteomic profiling using this method identified 1,507 unique proteins in overwintering and developing larvae of the mountain pine beetle, *Dendroctonus ponderosae* compared to the dozens to perhaps low hundreds of proteins that may be detected in 2-DE gel-based proteomics methods (Bonnett et al., 2012). Based on its high efficiency and sensitivity, iTRAQ has the potential to further advance research on the molecular mechanisms associated with diapause.

**Figure 1:**
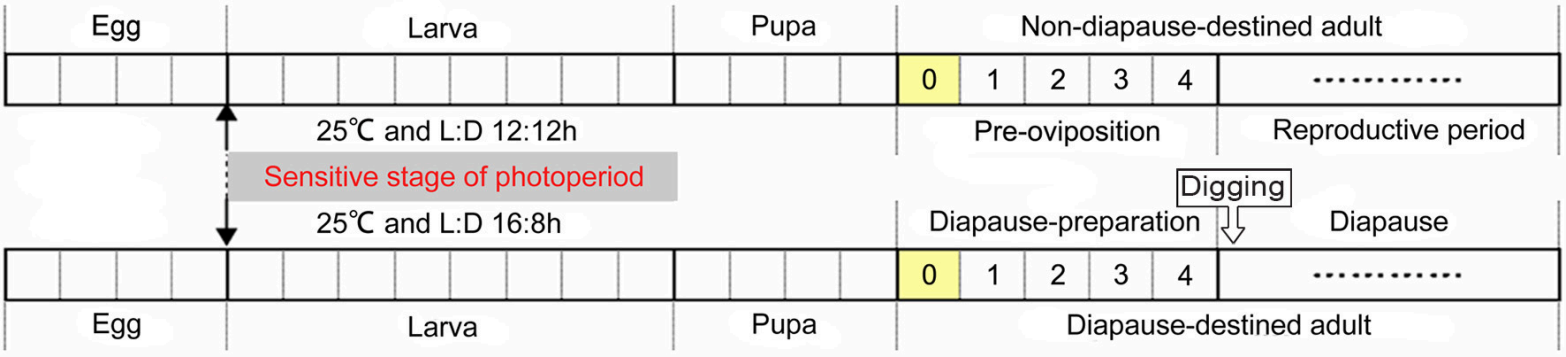
**Schematic life-cycle of the cabbage beetle *C*. *bowringi* under laboratory conditions at 25°C**. Each segment of the timelines indicates a single day of development (Xue et al., 2002a,b; Wang et al., 2004). Zero Represents the day at when female adults newly emerged and that had not yet had the opportunity to feed. One to four represents the days when female adults feed before either beginning reproduction or digging into the soil and entering diapause.

The cabbage beetle *Colaphellus bowringi* Baly is a serious pest of cruciferous vegetables in mountainous parts of China and some other Asian countries (Li et al., 2015). In response to photoperiodic and temperature cues this beetle enters reproductive diapause as adults that bury themselves in the soil during inclement periods in both the summer and winter seasons, with their peaks of reproductive and juvenile growth activities occurring in the spring and fall (Xue et al., 2002b; Wang et al., 2004). A population of these cabbage beetles from Xiushui, China enters diapause irrespective of photoperiod when the ambient temperature falls below 20°C, but does so in response to photoperiod (long day-length) at temperatures above 20°C (Xue et al., 2002b). Thus, cool temperatures cue the overwintering reproductive diapause, but when temperatures are warm long-day photoperiods cue the summer diapause. Although only adults enter diapause, the whole larval period is sensitive to photoperiod for summer diapause induction. Pre-diapause adults of both summer and winter diapause forms undergo a series of preparatory events after eclosion, including feeding for several days and digging into the soil, before entering diapause (Xue et al., 2002b; Wang et al., 2004). Diapause-destined female adults accumulate greater fat reserves than reproduction-destined females during this post-emergence adult diapause preparatory period (Tan et al., 2016). Thus, the photoperiodic diapause induction phase occurs during the larval stage and the diapause preparatory phase occurs primarily after adult emergence, making C. *bowringi* an excellent model for studying the diapause preparatory phase separately from the diapause induction phase (Figure 1).

In this paper, we present the results of experiments designed to investigate the diapause preparatory phase in the cabbage beetle. We used iTRAQ isobaric-tag-labeled proteomics, coupled with two-dimensional LC-MS/MS, to identify proteins and quantitatively compare relative protein abundance in the heads of newly emerged diapause-destined and non-diapause-destined adult female cabbage beetles. The head of insects contains the brain, compound eyes, corpus allatum and other important parts of the nervous, sensory, and endocrine systems where environmental cues related to diapause are perceived and processed, as well as muscle and cells of the anterior fat body (Denlinger, 2002; Koštál, 2006; Li et al., 2010; Zhang et al., 2012). It remains unclear how environmental signals such as photoperiod, that are perceived by larvae during the diapause induction phase are stored and subsequently regulate the major molecular processes involved in diapause preparation in cabbage beetle adults (Wang et al., 2004), but we focus on the head because we expect that it plays a critical role in this process.

In addition, we used RNA interference (RNAi) to investigate the function of a candidate protein that emerged from this screen for diapause preparation, fatty acid-binding protein (FABP). The results of our experiments demonstrated that FABP affects lipid accumulation, an important part of the diapause preparatory program. In addition to nutrient storage, increased stress hardiness is often associated with diapause preparation, and our proteomic data are consistent with this prediction. Surprisingly, our knock-down of FABP not only affected fat stores, but also decreased transcript abundance of two heat shock proteins, a class of molecules that have often been associated with increased stress hardiness in diapausing insects.

## Experimental section

### Ethics statement

The cabbage beetle *Colaphellus bowringi* Baly is a major pest of crucifers in mountainous areas of China. The field studies did not involve endangered or protected species, and no specific permissions were required for our research activities in these locations.

### Insects

Cabbage beetles, *C. bowringi*, were collected from the field in Xiushui County (29°1′N, 114°4′E), Jiangxi Province, China, and maintained in the laboratory since late November 2008 (Xue et al., 2002a; Ma et al., 2011). Experiments were conducted on the adult, female offspring of post-diapause adults that had emerged from the soil in early March 2013. All larvae and adults were kept in incubators (SPX–250IC; Boxun Medical Instruments, Shanghai, China) and fed on fresh radish leaves (*Raphanus stativus* var. *longipinnatus*) that were provided daily (Wang et al., 2004; Tan et al., 2016). Diapause-destined (DD) and non-diapause-destined (NDD) beetles were generated by rearing larvae under two different photoperiods; light:dark (L:D) 16:8, and L:D 12:12, at 25°C respectively (Figure 1) (Xue et al., 2002a,b; Wang et al., 2004). Sex was determined during the pupal stage (Wang et al., 2006) and only female adults were used in these experiments. The heads of newly emerged female adults (0 day) that had not yet had the opportunity to feed were collected and placed in 1.5 mL micro centrifuge tubes that were immediately frozen in liquid nitrogen and stored at −80°C. Six replicate samples, comprised of pools of at least fifty DD or NDD heads, were collected. Three of these replicates were used for protein identification and the other three for quantitative Real Time PCR (qRT-PCR). Meanwhile, three replicate groups of beetles were reared continuously at L:D 16:8 and L:D 12:12 to confirm their diapause status. The determination of diapause attributes were made after female adults feed for 7 days at 25°C in transparent plastic containers filled with soil in their respective photoperiod treatments. Diapausing beetles exhibit digging behavior, their ovarian development is arrested, and their abdomens clearly show their hypertrophied fat bodies (Xue et al., 2002b; Wang et al., 2004).

### Protein extraction and concentration determination

Head samples from cabbage beetles under each photoperiod were ground to a fine powder with a mortar and pestle under liquid nitrogen. Then, Lysis buffer containing phenylmethylsulphonyl fluoride (PMSF) (1 mM final concentration) and ethylenediaminetetraacetic acid (EDTA) (2 mM) was added, and after 5 min, 10 mM dithiothreitol (DTT) was added. The suspension was sonicated at 200 W for 15 min and then centrifuged for 20 min at 4°C, 25,000 × g. The supernatant was mixed well with 5 × volume of chilled acetone and incubated at −20°C overnight. After centrifugation for 20 min at 4°C, 16,000 × g, the supernatant was discarded. The precipitate was washed with chilled acetone three times. The pellet was air-dried and dissolved in Lysis buffer (7M urea, 2 M thiourea, 4% NP-40 Nonidet (NP-40), 20 mM Tris-HCl, pH 8.0–8.5). The suspension was sonicated at 200 W for 15 min and centrifuged for 20 min at 4°C, 25,000 × g. The supernatant was transferred to another tube. Protein disulphide bonds were reduced by treatment with 10 mM DTT at 56°C for 1 h and the cysteine was blocked by using 55 mM iodoacetamide in a dark room for 45 min. Five times the volume of the supernatant in chilled acetone was added and incubated for 2 h at −20°C. After centrifugation for 20 min at 25,000 × g, the supernatant was discarded, and the pellet was air-dried for 5 min. The pellet was dissolved in 200 μL 0.5 M tetraethyl-ammonium bicarbonate and sonicated for 15 min. Samples were centrifuged for 20 min at 25,000 × g and the protein concentration of the supernatant was measured by the Bradford method (Bradford, 1976). Then, a volume containing 100 μg of protein was taken out from each sample solution and treated with Trypsin Gold with the ratio of protein: trypsin = 20:1 to digest the proteins by incubating for 4 h at 37°C. Because the initial 4 h digestion step did not produce complete fragmentation, we additionally exposed the partially digested proteins again to Trypsin Gold with the ratio of protein: trypsin = 20:1, continuing digestion for an additional 8 h at 37°C.

### iTRAQ labeling

After trypsin digestion, peptides were labeled with iTRAQ reagents. Briefly, desalted samples were reconstituted in 30 μL of dissolution buffer and mixed with two different isobaric tags. Peptides from the heads of DD individuals and heads of NDD individuals were labeled with tags of mass 115 and 116 respectively using the iTRAQ Reagent 8-plex Kit according to the manufacturer's protocol. Labeled samples were pooled together and dried using a speedvac.

### Strong cation exchange (SCX) chromatography

For SCX chromatography we used a Shimadzu LC-20AB high performance liquid chromatography (HPLC) system where peptides from the digestion step were reconstituted with 4 mL of buffer A (25 mM NaH_2_PO_4_ in 25% acetonitrile (ACN), pH 2.7) and loaded onto a 4.6 × 250 mm Ultremex SCX column containing 5-μm particles (Phenomenex). Peptides were eluted at a flow rate of 1 mL/min with a gradient of buffer A for 10 min, 5–60% buffer B (25 mM NaH_2_PO_4_, 1 M KCl in 25% ACN, pH 2.7) for 27 min, and then 60–100% buffer B for 1 min. The system was then maintained in 100% buffer B for 1 min before equilibrating with buffer A for 10 min prior to the next injection. Elution was monitored by measuring absorbance at 214 nm, and fractions were collected every 1 min. Eluted peptides were pooled into 20 distinct fractions, desalted with a Strata X C18 column (Phenomenex), and vacuum-dried.

### LC-ESI-MS/MS analysis

Each fraction was resuspended in buffer A (5% ACN, 0.1% formic acid) and centrifuged at 20,000 x g for 10 min. In each fraction, the final concentration of peptides was ~0.5 μg/μL. Ten μL of supernatant was loaded on an Shimadzu LC-20AD nanoHPLC by an autosampler onto a 2 cm C18 trap column (inner diameter 200 μm) and the peptides were eluted onto a custom 10 cm analytical resolving C18 column (inner diameter 75 μm) made in-house. The samples were loaded at 8 μL/min for 4 min, then the 35 min gradient was run at 300 nL/min starting from 2 to 35% buffer B (95% ACN, 0.1% FA), followed by 5 min linear gradient to 60%, then, followed by 2 min linear gradient to 80%, and maintenance at 80% B for 4 min, and finally returning to 5% B in 1 min.

Data acquisition was performed with a Triple TOF 5600 System (AB SCIEX, Concord, ON) fitted with a Nanospray III source (AB SCIEX, Concord, ON) and a pulled quartz tip as the emitter (New Objectives, Woburn, MA). Data were acquired using an ion spray voltage of 2.5 kV, curtain gas of 30 PSI, nebulizer gas of 15 PSI, and an interface heater temperature of 150°C. The MS was operated with a RP of greater than or equal to 30,000 FWHM for TOF MS scans. For IDA, survey scans were acquired in 250 ms and as many as 30 product ion scans were collected if exceeding a threshold of 120 counts per second (counts/s) and with a 2+ to 5+ charge-state. Total cycle time was fixed to 3.3 s. The Q2 transmission window was 100 Da for 100%. Four time bins were summed for each scan at a pulse frequency value of 11 kHz by monitoring the 40 GHz multichannel TDC detector with four-anode channel detections. A sweeping collision energy setting of 35 ± 5 eV adjusted rolling collision energy was applied to all precursor ions for collision-induced dissociation. Dynamic exclusion was set for 1/2 of the peak width (15 s), and then the precursor was refreshed off the exclusion list.

### Protein identification and quantification statistical analysis

Mascot software (version 2.3.02, Matrix Science, London, UK) was used to identify and quantify proteins. Only unique peptides were selected for quantification. After proteins had been quantified, the *de novo* transcriptome database of *C. bowringi* deposited into the NCBI Sequence Read Archive (Accession: SRP026471) (Tan et al., 2015), NCBI *Tribolium castaneum* database (54364 seqs, download from http://www.ncbi.nlm.nih.gov/protein?term=txid7070%5BOrganism%5D&cmd=search), NCBInr, SwissProt, and UniProt, were all searched for matches. The MGF (Mascot generic format) search file consisted of Spectra from 10 fractions. We parameterized searches to include single missed cleavages with trypsin; carbamidomethylation at Cys and oxidation at Met selected as fixed modifications and variable modifications, respectively. The tolerances for peptides and MS/MS were set at 10 ppm and 0.05 Da, respectively. Filters were set as follows: significance threshold *P* < 0.05 (with 95% confidence) and ion score or expected cut-off less than 0.05 (with 95% confidence) for protein identification. The setting “median” was chosen for protein ratio type (http://www.matrixscience.com/help/quant_config_help.html); minimum precursor charge was set to 2+, and the minimum peptides value was set to 2 with only unique peptides used to quantify proteins. Median intensities were used for normalization and outliers were removed automatically. The peptide threshold was set as above for identity. We only used ratios with p < 0.05, and only fold changes of ≥1.2 were considered as significant.

### Quantitative real-time PCR (qRT-PCR) analysis of transcript abundance

qRT-PCR analysis was used to quantify transcript abundance for target proteins. Total RNA was extracted using TRIzol (TaKaRa Bio., Dalian, China) following the manufacturer's protocol. Concentration and purity of the total RNA isolated from different samples were determined using a NanoDrop 2000 (Thermo Scientific, Wilmington, DE, USA). One microgram of total RNA was used to synthesize first-strand cDNA using the PrimeScriptRT reagent kit with gDNA Eraser (Perfect Real Time) (TaKaRa Bio, Dalian, China), according to the manufacturer's protocol. Synthesized cDNA was stored at −20°C. Quantitative PCR reactions were performed using a MyIQ2 Two-color Real-time PCR Detection System (Bio-Rad, USA) and SYBR Premix Ex Taq II (TaKaRa, Dalian, China). *Ribosomal protein L19* (*RPL19*) was amplified for internal standardization based on our previous evaluation of reference genes in *C. bowringi* (Tan et al., 2015). Primers for gene amplification used in qRT-PCR are shown in Table S1. cDNA samples were derived from each of the three independent biological replicates for each experimental condition, and each biological replicate was analyzed with three technical replicates. Relative qRT-PCR expression data were calculated using the 2^−ΔΔCT^ method (Schmittgen and Livak, 2008). Eight proteins, namely FABP (Gerstner et al., 2008), alcohol dehydrogenase (Fan et al., 2013), U6 snRNA-associated Sm-like protein LSm3, protein phosphatase 1 (Gonsalvez et al., 2008), catalytic subunit (Bao and Xu, 2011), cytoglobin-1-like (Hoogewijs et al., 2007), tricarboxylate transport protein (Dwivedi et al., 2014), dolichyl-diphosphooligosaccharide (Bao et al., 2010), and protein disulfide isomerase (Filonova et al., 2013), were selected for verifying the protein expression patterns.

### Functional classification of proteins

The gene function databases complied for the Gene Ontology (GO), Cluster of Orthologous Groups of proteins (COG), and the Kyoto Encyclopedia of Genes and Genomes (KEGG) pathways were used to infer putative functions of identified proteins. Detailed information can be found on http://www.geneontology.org. COG is the database for orthologous protein classification. We compared identified proteins with the COG protein database to predict the function of proteins and conduct statistical analyses. For KEGG pathways (Kanehisa et al., 2004), we deduced the main biochemical metabolism and signal transduction pathways of identified proteins by pathway analysis. Hypergeometric tests were applied to assess the significance of over-representation for predefined protein sets (*p* ≤ 0.05).

### RNA interference

A 321-bp fragment of *C. bowringi FABP* was amplified by PCR using the corresponding primers (Table S1) and purified with phenol-chloroform. One microgram of PCR product was used as the template for double-stranded RNA (dsRNA) synthesis using the T7 transcription kit (Fermentas, Lithuania) according to the manufacturer's protocol. dsRNA was precipitated in ethanol overnight, resuspended in RNase-free water, and quantitated at 260 nm using a NanoDrop 2000 Spectrophotometer (Thermo Fisher Scientific Inc.) before microinjection. The quality and integrity of dsRNA were determined by agarose gel electrophoresis. Microinjection was performed using a Nanoliter 2010 microinjection system (Word Precision Instruments INC, Sarasota, FL 34240-9258 USA). The dsRNA of *green fluorescent protein* (dsGFP) was used as the control. Two microgram of either FABP or GFP dsRNA in 200 nL was microinjected into the ventral abdomen of each newly emerged female adult before they had fed (day 0). Total RNA from the fat body or head was extracted after 2 and 4 days post-treatment to determine whether RNAi treatments affected *FABP* or heat shock protein transcript abundance. After 7 days post-treatment, the survival rates and diapause incidence were determined for beetles in this study.

### Fat body morphology

The fat body was dissected from individual beetles in a Petri dish on wax containing phosphate buffer solution (PBS) (Zhou et al., 2010) and photographed with a digital camera (Nikon D5100, Nikon Imaging (China) Sales, China) mounted on a stereo-microscope (SMZ-t4, Chong Qing Optec Instrument, China).

The number of lipid droplets in fat body cells were visualized after Oil Red O staining that can be used to quantify fat concentration by staining intracytoplasmic lipids (Ramirez-Zacarias et al., 1992; Koopman et al., 2001; Singh et al., 2009). Fat bodies were fixed in 4% paraformaldehyde solution for over 24 h at 4°C and the fixed tissues were then cut into 8–10 μm thick sections at −20°C (Tissue-Tek; Sakura Finetechnical, Tokyo, Japan) and mounted on slides. The slides were air dried for 10 min at room temperature, fixed in ice cold 4% paraformaldehyde for 15 min, and then washed 3 times with PBS. Slides were incubated in pre-warmed Oil Red O solution for 10–15 min and again washed 3 times with PBS. Slides were then stained in Oil Red O solution for 1–2 h in a 37°C oven, differentiated in 75% alcohol solution for 2 s, and washed with distilled water for 1 min. Slides were then counterstained for 1–2 min in Harris hematoxylin, rinsed in tap water and differentiated in 1% hydrochloric acid alcohol solution for a few seconds. After mounting in glycerogelatin, sections were observed under a phase contrast microscope (Nikon eclipse CI). Lipid droplets in adipocytes stained red whereas cell nuclei stained blue. Lipid droplets were quantified by measuring the intensity of the red signal using ImageJ software (Schneider et al., 2012).

### Data analysis

One-way analysis of variance (ANOVA) was used to evaluate *FABP* and heat shock protein transcript abundance. Differences between means were examined using Tukey's Least Significant Difference (LSD) test. Differences in transcript abundance and lipid droplet content were evaluated using the independent-samples *t*-test. *P* < 0.05 were considered statistically significant.

## Results

### Identification of proteins with iTRAQ

Under diapause-inducing conditions (L:D 16:8 and 25°C), 100% of DD females entered diapause, these diapausing beetles dug into the soil, their ovarian development was arrested, and their abdomens were full of hypertrophied fat bodies. At the same time, 8.9% of NDD females entered diapause under conditions not expected to induce diapause (L:D 12:12 and 25°C). Three replicate iTRAQ-LC-MS/MS assays identified a total of 3,175 unique proteins (confidence > 95%) from 62,030 spectra, of which 9,512 peptides were unique (Table 1). The full list of these proteins is available in Table S2. Comparison with the GO database indicated that 23, 16, and 14 protein groups could be assigned to the Biological process (BP), Cellular component (CC) and Molecular function (MF) categories, respectively (Figure S1). Among these categories, cellular process, cell part, and protein binding were the most abundant sub-categories in BP, CC, and MF respectively.

**Table 1:** **Proteins identified in the head of female adult *C. bowringi***.

**Items**	**Number**
Total spectra	1,030,260
Spectra	62,030
Unique spectra	51,737
Peptide	10,262
Unique peptide	9,512
Protein	3,175

### Quantification and statistical analysis of identified proteins and qRT-PCR validation

We identified 297 differentially expressed proteins (9.4% of total proteins ≥1.2-fold change, *P* < 0.05) (Table S3), 141 of which were up-regulated and 156 were down-regulated in the heads of DD females.

To verify these apparent differences in protein expression, we used qRT-PCR to analyze transcript abundance of 8 selected proteins in the heads of another cohort of cabbage beetles.

**Figure 2:**
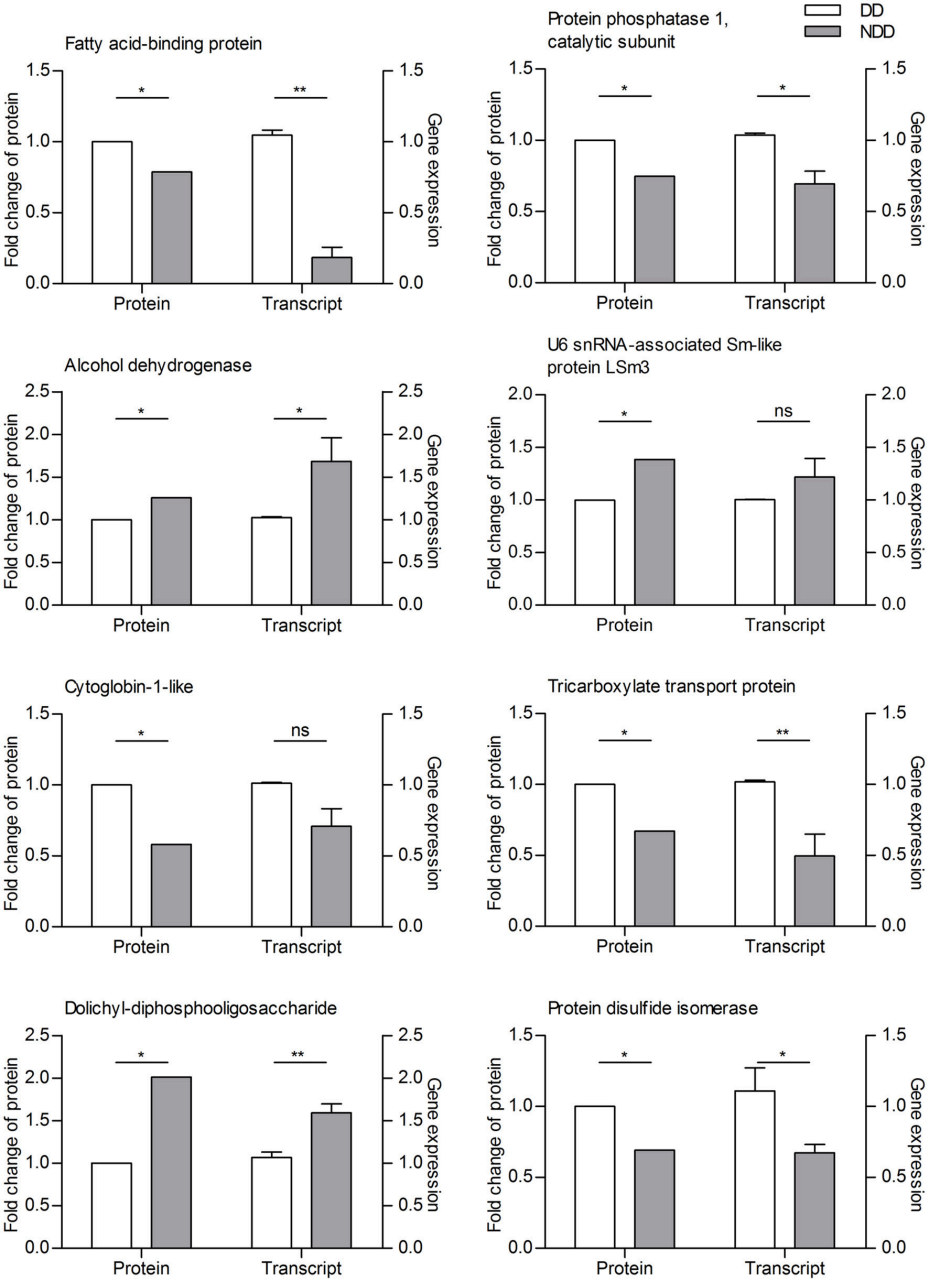
**Correlation of the expression patterns between proteins and transcripts as determined by iTRAQ and qRT-PCR on three replicate samples of adult female *C*. *bowringi* heads**. DD, diapause-destined; NDD, non-diapause-destined. The samples used for iTRAQ and qRTPCR experiments were from distinct batches of heads. ^**^P < 0.01, ^*^P < 0.05, ns, not significant (*t*-test).

For proteins that were in greater abundance in diapause-destined females, *FABP, protein phosphatase 1, catalytic subunit, tricarboxylate transport protein*, and *protein disulfide isomerase*, all seven showed higher transcript abundance. For the three proteins we selected that were down-regulated in diapause-destined females, *alcohol dehydrogenase* and *dolichyl-diphosphooligosaccharide*, both had lower transcript abundance as expected, but transcript abundance for *U6 snRNA-associated Sm-like protein LSm3* was not detectably higher in females not destined for diapause, although there was a trend toward higher transcript abundance in females not destined for diapause that is consistent with our proteomic data (Figure 2).

### Functional classification of differentially expressed proteins

**Figure 3:**
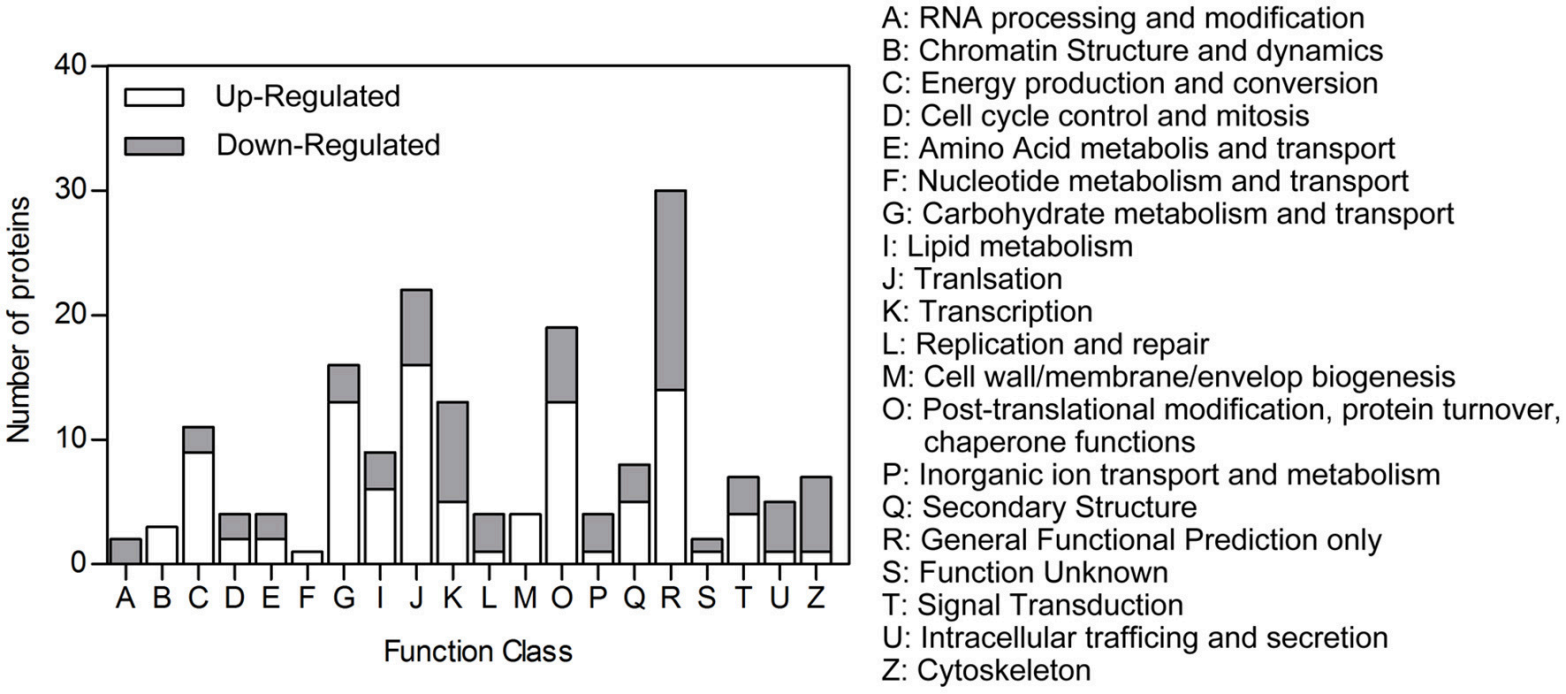
**COG function classification**. Peptides that were differentially expressed in the heads of diapause-destined vs. non-diapause-destined female *C. bowringi* beetles were mapped to protein orthologs and assigned putative functions based on the Clusters of Orthologous Groups of proteins database. Many of the categories of proteins that were up-regulated in diapause-destined females were metabolism and engeretics, as may be expected for individuals packing on additional nutrient stores during the diapause preparatory period.

Of the differentially expressed proteins we identified, 138 could be classified in the COG database. Among these, 82 up-regulated proteins could be assigned to 19 COG categories and 56 down-regulated proteins to 17 COG categories (Figure 3). Many abundant up-regulated proteins in the heads of DD females were involved in energy production and conversion, carbohydrate metabolism and transport, and lipid metabolism (Table 2, Table S3). This, in turn, suggests that many energetic metabolic pathways are vigorously triggered even early during diapause preparation.

**Table 2:** **The top six statistics of pathways enrichment for differentially expressed proteins in COG function classification**.

**COG code**	**COG functional-categories**	**Number**
**UP-REGULATED DIFFERENTIALLY EXPRESSED PROTEINS UNDER DIAPAUSE-DESTINED FEMALE HEAD**		
J	Tranlsation	16
R	General Functional Prediction only	14
**G**	**Carbohydrate metabolism and transport**	**13**
O	Post-translational modification, protein turnover, chaperone functions	13
**C**	**Energy production and conversion**	**9**
**I**	**Lipid metabolism**	**6**
**DOWN-REGULATED DIFFERENTIALLY EXPRESSED PROTEINS UNDER DIAPAUSE-DESTINED FEMALE HEAD**		
R	General Functional Prediction only	16
K	Transcription	8
J	Tranlsation	6
O	Post-translational modification, protein turnover, chaperone functions	6
Z	Cytoskeleton	6
U	Intracellular trafficing and secretion	4

*The pathways related to energetic metabolic are highlighted as bold*.

Because lipid metabolism pathways are particularly important during the diapause preparation phase (Sim and Denlinger, 2009; Hahn and Denlinger, 2011), we matched differentially expressed protein sequences to the reference canonical lipid metabolism pathways in the KEGG database to identify potential diapause-associated proteins involved in lipid metabolism. A total of 9 proteins could be assigned to 6 lipid metabolic pathways, of which 7 proteins were up-regulated in the heads of DD females (Table 3). Interestingly, we found that FABP was assigned to both the peroxisome proliferator-activated receptor (PPAR) signaling pathway, which can regulate lipid metabolism, and the fat digestion and absorption pathway. In addition, when mapping to vertebrate proteins, the beetle FABP was assigned to both FABP1 (liver) and FABP7 (brain) in the PPAR signaling pathway (Schoonjans et al., 1996; Gilde et al., 2003). This suggests that lipid metabolism pathways are upregulated, and that FABP may regulate lipid metabolism, during the onset of diapause preparation.

**Table 3:** **Differentially expressed proteins assigned to lipid metabolism pathways in KEGG function classification**.

**Pathway**	**Accession**	**KO**	**Definition**	**Fold change (*p* < 0.05)**
PPAR signaling pathway	**Unigene1588**	**K08750**	**Fatty acid-binding protein 1, liver**	**0.787**
	**Unigene1588**	**K08756**	**Fatty acid-binding protein 7, brain**	**0.787**
	CL538.Contig1	K08766	Carnitine O-palmitoyltransferase 2	1.393
Fatty acid elongation	Unigene3275	K00022	3-hydroxyacyl-CoA dehydrogenase	0.748
Fatty acid metabolism	Unigene3275	K00022	3-hydroxyacyl-CoA dehydrogenase	0.748
	CL538.Contig1	K08766	Carnitine O-palmitoyltransferase 2	1.393
Fat digestion and absorption	**Unigene1588**	**K08750**	**Fatty acid-binding protein 1, liver**	**0.787**
	gi|189238450	K14462	Apolipoprotein B	0.791
	CL3359.Contig1	K14463	Microsomal triglyceride transfer protein large subunit	1.616
Glycerophospholipid metabolism	gi|91083661	K00111	Glycerol-3-phosphate dehydrogenase	0.821
Glycerolipid metabolism	CL3010.Contig1	K01189	Alpha-galactosidase	0.667

*Fold change <0.83 indicates proteins that were in greater abundance in the heads of diapause-destined female adults. Fold change >1.2 indicates the protein that were in greater abundance in the heads of non-diapause-destined female adults. The definitions related to fatty acid-binding protein are highlighted as bold*.

Beyond automated enrichment of functional groups and our interrogation of lipid metabolism, we also inspected the lists of differentially expressed proteins to detect additional patterns. One apparent difference is that non-diapause destined individuals had greater abundance of cuticular proteins expressed, perhaps indicating a trajectory of faster post-emergence growth than in the diapause-destined individuals. Inspection of proteins significantly more abundant in the heads of diapause-destined females revealed several previously associated with increased stress hardiness including a putative small heat shock protein, a putative peptidoglycan recognition protein that could be involved in increased pathogen resistance, as well as glutathione S-transferase and thioredoxin, two proteins that have been implicated in ameliorating both oxidative stress and xenobiotic stress. Similar processes of increased hardiness to pathogens, oxidative stress and heat shock protein expression have commonly been found in other “omics” studies of insect diapause (Ragland et al., 2010; Poelchau et al., 2013; King and MacRae, 2015; Poupardin et al., 2015; Qi et al., 2015; Meyers et al., 2016 as some examples). Two proteins that stand out as being associated with development of synaptic activity in nervous systems, bruchpilot and syntaxin (Broadie et al., 1995; Wagh et al., 2006; Short, 2013), were also more abundant in diapause-destined females and have previously been associated with diapause in other insects and *C. elegans* worms (Ailion and Thomas, 2003; Ragland et al., 2011). Last, a protein that stood out as being more abundant in diapause-destined females is a putative farnesoic acid o-methyltransferase, the enzyme that catalyzes the last step in juvenile hormone synthesis (Shinoda and Itoyama, 2003; Noriega, 2014).

### Effect of *FABP* suppression on fat body hypertrophy, lipid droplet content, and heat shock protein transcript abundance

**Figure 4:**
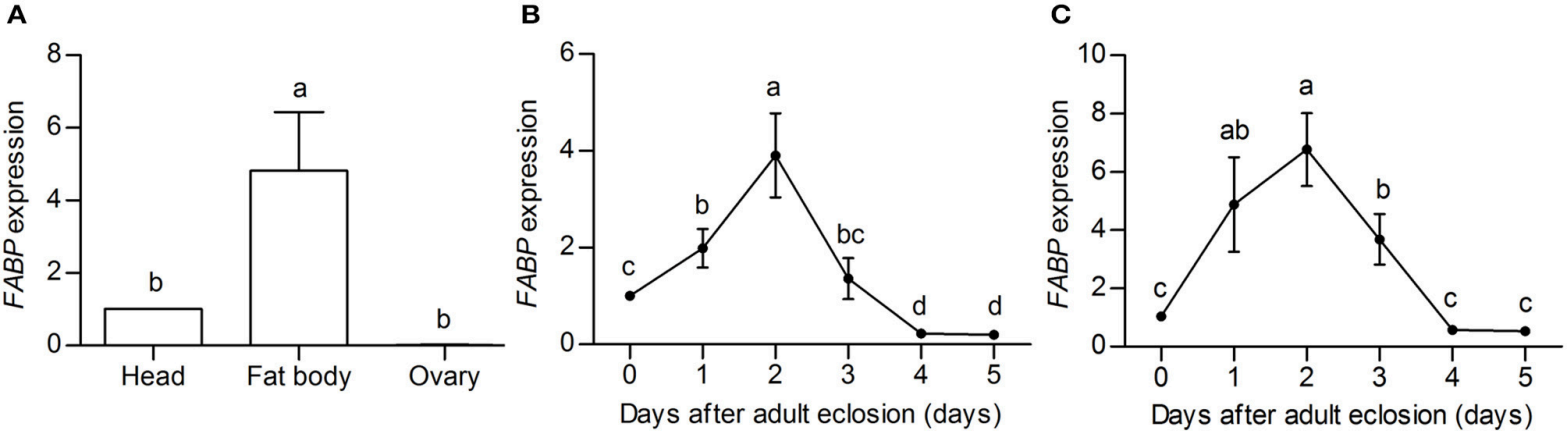
***FABP* expression profiles obtained by qRT-PCR on three replicate samples of diapause-destined adult female *C. bowringi* heads. (A)** Spatial expression of *FABP* in different tissues. **(B)** Temporal expression of *FABP* in the fat body after adult eclosion. **(C)** Temporal expression of *FABP* in the head after adult eclosion. Day 0 indicates the day on which female adults closed, but had not yet had the opportunity to feed; days 1–5 indicate the number of days in which females fed post-eclosion. Different letters indicate significant difference in the *FABP* expression levels (*P* < 0.05, ANOVA followed by LSD test).

Because FABP peptides were clearly more abundant in DD females than that in NDD females, we examined spatial patterns of *FABP* transcript abundance in the head, fat body (the adipose tissue and liver analog of insects), and ovaries of DD females to investigate the function of FABP in diapause preparation. The results showed that *FABP* transcripts were more highly expressed in the fat body and head than in the ovary (Figure 4A). We then examined the temporal pattern of *FABP* transcript abundance in the head and fat body during the diapause preparation phase. *FABP* transcript abundance in both the head and the fat body increased with feeding, peaked 2 days after eclosion, and then declined on day 4 after eclosion (Figures 4B,C). These results suggested that *FABP* may play an important role during diapause preparation, specifically considering that *FABP* transcript abundance increases at the same time in the diapause preparatory period that we have previously shown fat is rapidly accumulated (Tan et al., 2016, Figure 1). *FABP* transcript abundance also declined coincident with the time in the diapause preparatory period that we have previously shown fat storage plateaus just prior to the time in which beetles have stopped feeding and dig themselves into the soil to enter diapause (Tan et al., 2016, Figure 1).

**Figure 5:**
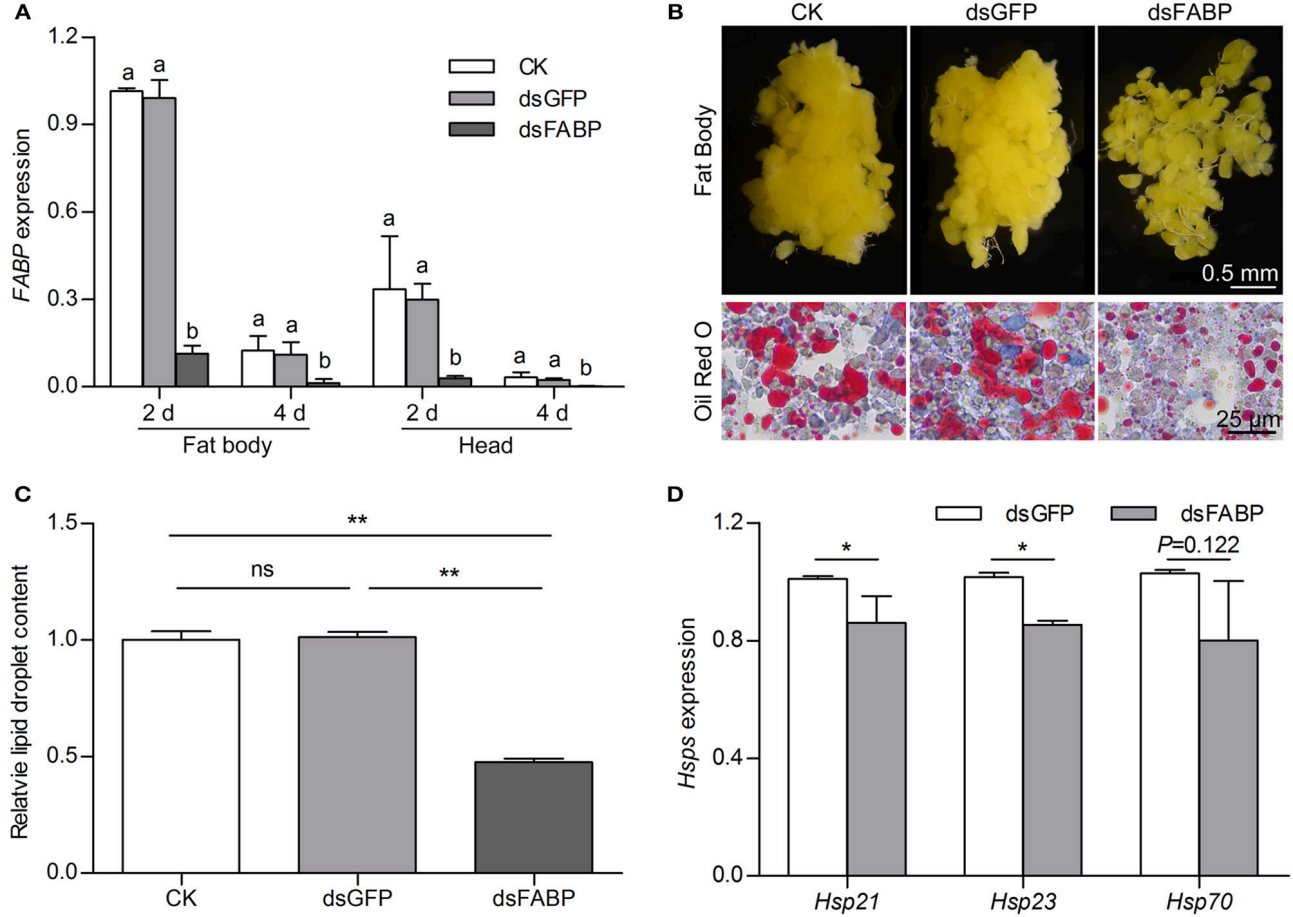
**Effects of *FABP* RNAi on *FABP* transcript abundance (A)**, fat body morphology **(B)**, lipid droplet accumulation **(C)** and heat shock protein transcript abundance **(D)**, in diapause-destined adult female *C. bowringi*. *FABP* expression in the fat body and head were determined by qRT-PCR on three replicate samples 2 and 4 days after RNAi injection on the day of adult eclosion. Different letters indicate significant difference in *FABP* expression (*P* < 0.05, ANOVA followed by LSD test). Fat body morphology was examined in DD females 4 days after injection; CK = control check group, dsGFP = group with dsRNA of *green fluorescent protein* injection, dsFABP = RNAi treatment group. Cellular lipid droplet accumulation was detected using Oil Red O staining and the relative number of lipid droplets quantified using ROI Manager in ImageJ. The mean was the average of the optical density of 8 randomized areas. ^**^P < 0.01, ns = not significant (*t*-test). Relative transcript abundance of three different heat shock proteins in the fat body was determined 4 days after RNAi injection. ^*^P < 0.05 (*t*-test).

To investigate the potential function of FABP in diapause preparation, we silenced it by injecting *FABP* dsRNA (ds*FABP*) into newly emerged DD females that had not yet had the opportunity to feed (0 day) and measured *FABP* transcript abundance in the head and fat body at 2 and 4 days after ds*FABP* injection. qRT-PCR showed that *FABP* transcript abundance in the ds*FABP* treatment group was significantly lower in both the head and fat body than that in a control check (CK) or ds*GFP* group (Figure 5A). In addition, the survival rates of the different treatment groups at 7 days post-dsRNA injection were all above 95%. The diapause incidence of the CK, ds*GFP*, and ds*FABP* groups were 91.15, 90.75, and 89.83%, respectively. Therefore, knockdown of *FABP* did not affect either survival or diapause incidence of DD *C. bowringi*.

A previous study by our group showed that DD beetles accumulate greater fat than NDD beetles during the diapause preparation phase (Tan et al., 2016). In this study, photomicrographs revealed hypertrophy and clumping of the fat body in the CK and ds*GFP* groups at 4 days after dsRNA injection. Hypertrophy of fat body was, however, inhibited in the ds*FABP* group and the fat body became detached (Figure 5B). Suppressing *FABP* transcript abundance during diapause preparation substantially decreased lipid accumulation. We further investigated the effect of *FABP* suppression on lipid droplet accumulation with Oil Red O staining. Red-stained lipid droplets were significantly lower in the ds*FABP* group than that in the CK and ds*GFP* groups (Figures 5B,C), suggesting that accumulation of lipid droplets was reduced by *FABP* transcript suppression.

Because most insects do not feed during the long diapause period, increased lipid stores are often associated with hardiness against metabolic stress (Arrese and Soulages, 2010; Hahn and Denlinger, 2011). In addition to metabolic stress, diapausing insects also face thermal stress, desiccation, and other environmental challenges (MacRae, 2010; Storey and Storey, 2012; Colinet et al., 2015). Heat shock proteins are known regulators of environmental stress and are often associated with increased stress hardiness in diapausing insects (Rinehart et al., 2007; Gkouvitsas et al., 2008, 2009). Here, we found that knocking down *FABP* not only affected fat accumulation, but also *Hsp*s. Transcript abundance of *Hsp21* and *Hsp23* in the fat body decreased at 4 days after FABP dsRNA injection. *Hsp70* transcript abundance trended toward decreasing but the effect was not statistically significant (Figure 5D). These results indicate that *FABP* suppression affects both lipid accumulation and transcript abundance of two heat shock proteins during diapause preparation.

## Discussion

Understanding the molecular changes associated with the diapause preparation phase is important for understanding regulation of insect diapause and also understanding the consequences of those preparatory mechanisms for surviving diapause and performing in the post-diapause period (Denlinger, 2002; Hahn and Denlinger, 2011). We found a total of 297 differentially expressed proteins in the heads of diapause-destined and non-diapause-destined females. Although proteomics has been used to investigate diapause several times, only two previous proteomic studies focused on diapause preparation (Zhang et al., 2012, 2013). One, a proteomic analysis of the *H. armigera* larval brain, using a 2-DE approach detected 49 differentially expressed proteins, 37 of which were successfully identified using MALDI-TOF/TOF mass spectrometry (Zhang et al., 2012). The other study used the same techniques to detect 37 differentially expressed proteins in *H. armigera* larval hemolymph, 28 of which were successfully identified (Zhang et al., 2013). Using the iTRAQ technique in combination with LC-MS/MS, we identified 3,175 unique proteins in the head of adult female *C. bowringi*, of which 297 were significantly differentially expressed between DD and NDD females. As a step to confirm the observed patterns, we examined transcript abundance of 8 of our differentially expressed proteins as has been done to confirm patterns of differential abundance in other proteomics studies (Yang et al., 2013; Muñoz-Gómez et al., 2014; Zhang et al., 2015). From our transcriptional assays, 7 of the 8 were in clear agreement with our iTRAQ peptide counts and the last one was trending in the correct direction. Thus, we have good confidence in the proteomic patterns represented here.

To build hypotheses about the probable functions of differentially expressed proteins, we classified them according to commonly used protein function classification systems. 138 of the 297 differentially expressed proteins identified in the heads of adult female *C. bowringi* could be assigned to 20 COG categories. Of these, 41 (almost 30%) were assigned to categories associated with energy production and conversion, carbohydrate metabolism and transport, lipid metabolism, and other metabolic pathways; and 7 were assigned to the signal transduction category. The functional classification suggests that most proteins recovered in this study may come from tissues other than brain, with head fat-body cells and muscle perhaps driving the majority of the proteomic signal.

Many abundant, up-regulated proteins in diapause-destined females were related to energy production and conversion, carbohydrate metabolism and transport, and lipid metabolism. These observations are consistent with both our own data showing that diapause-destined cabbage beetles accumulate greater fat stores during the diapause preparatory period (Tan et al., 2016), and with many other reports that accumulation of nutrient stores is an important function of the diapause preparatory period (Koštál, 2006; Hahn and Denlinger, 2007; Sim and Denlinger, 2009; Reynolds et al., 2012; Zhang et al., 2012, 2013). FABP, which was significantly up-regulated during the onset of diapause-preparation phase in diapause-destined females, appears to be critical for proper accumulation of fat stores during diapause preparation.

FABP is primarily considered a fatty acid transport protein, and thus is most likely acting to support increased lipid accumulation in the preparation phase of diapause-destined female beetles (Haunerland et al., 1993; Zhang and Haunerland, 1998; Arrese and Soulages, 2010). In agreement with this concept, we found that *FABP* had higher mRNA abundance in the abdominal fat body and head compared to the ovary, and that mRNA abundance peaked after newly emerged adult females had fed for 2 days. The timing of this peak in *FABP* transcript abundance coincided with peak timing of fat accumulation in diapause-destined *C. bowringi* (Tan et al., 2016). In previous work, we showed that diapause-destined females mainly directed nutrients into triacylglycerol in the fat body, whereas reproduction-destined females primarily allocated nutrients to ovarian development, and that these differences are apparent after female adults had fed for 2 days (Tan et al., 2016). The fat body plays a major role in energy storage and use during diapause in insects (Hahn and Denlinger, 2007; Arrese and Soulages, 2010). Previous studies have shown that extreme lipid deposition could cause hypertrophy of the fat body and storage of large quantities of lipid reserves in adipocytes as cytoplasmic lipid droplets (Mitchell and Briegel, 1989; Bowen, 1992; Sim and Denlinger, 2009; Arrese and Soulages, 2010). We found that silencing *FABP* with RNAi restricted lipid accumulation in diapause-destined individuals, inhibiting hypertrophy of the fat body, and caused the fat body cells to become detached and contain reduced numbers of lipid droplets. This suggests that *FABP* plays a critical role in the accumulation of nutrients required for diapause preparation.

Beyond its role in fatty acid transport however, FABP has also been implicated as a signaling molecule in some contexts. Bioinformatic analysis suggested that the up-regulated FABP protein we identified in the heads of diapause-destined females also had some similarity to vertebrate FABPs that have been associated with peroxisome proliferator-activated receptor (PPAR) signaling (e.g., FABP7, Table 3). PPARs are a family of nuclear hormone receptors that act as transcription factors and are well known to regulate aspects of metabolism, feeding and appetitive behavior, as well as immunity, growth, and cellular proliferation (Chmurzyńska, 2006). For example, *FABP7* mRNA levels have been shown to be involved in sleep and activity mechanisms in the adult rodent brain where they are higher during exposure to light than when in darkness (Gerstner et al., 2008), and also similarly affect sleep and memory in *Drosophila* (Gerstner et al., 2011). Perhaps FABP expression observed during diapause preparation could be related to photoperiodic timing?

It is also possible that the increased abundance of FABP in the heads of diapause-destined adult female *C. bowringi* could affect other signaling molecules important to diapause. FABPs are known to carry other lipophilic substances such as eicosanoids and retinoids in vertebrate systems (Jordanova et al., 2009), and it has been supposed that they could bind to insect juvenile hormone (Newman et al., 2005). Suppression of juvenile hormone production is a common trait of adult reproductive diapause across many insect species (Denlinger, 2002). Although, suppression of juvenile hormone signaling has not been fully confirmed for *C. bowringi* adult reproductive diapause, it seems likely to be important (Liu et al., 2016). Interestingly, another protein that was more abundant in diapause-destined females, farnesoic acid o-methyltransferase, is a critical enzyme involved in juvenile hormone synthesis that is known to play an important role in regulating some developmental transitions in insects (Shinoda and Itoyama, 2003; Noriega, 2014). Thus, it is possible that our observed FABP plays some role in regulating activities in the diapause preparatory period through interactions with juvenile hormone signaling. However, knockdown of FABP did not affect the propensity of diapause-destined females to enter diapause in our study.

Because our proteomic screen used the whole heads of female beetles upon emergence, very early in the diapause preparation phase, we cannot distinguish whether our candidate FABP protein was localized to the region of the brain with PPAR signaling activity, to fat body tissue in the head surrounding the brain, or some other tissue contained in the head like developing mandibular muscle. Interestingly, work in *Drosophila* has shown that fat body cells in the adult head differ substantially in both protein composition and function from fat body cells in the abdomen (Benes et al., 1996; Fujii and Amrein, 2002). Fat body cells in the head in particular have been implicated in signaling roles that may affect reproduction and longevity, two processes also correlated with adult reproductive diapause (Hwangbo et al., 2004). Further work on localization of FABP proteins in the heads of diapause-destined female beetles as well as additional work manipulating both PPAR signaling and fatty acid transport and lipid biosynthesis will be needed to test between the alternative hypotheses that FABP either (1) plays a regulatory role to control fattening in the brain, (2) is critical for transporting fatty acids during fat anabolism, or (3) some combination of both.

In addition to proteins associated with metabolism that were nominated by our enrichment analyses, directly examining lists of differentially expressed proteins in our study also showed greater abundance of several proteins that have been implicated in increased stress hardiness in diapause-destined females including heat shock proteins, immune proteins, and antioxidants (Table S3). Increased stress hardiness is commonly a facet of diapause in many insect species and transcripts or proteins associated with stress hardiness have been identified in many studies (MacRae, 2010; Ragland et al., 2010; Poelchau et al., 2013; Poupardin et al., 2015; Qi et al., 2015; Meyers et al., 2016). Heat shock proteins in particular are associated with stress hardiness and are often highly expressed in diapausing individuals (Rinehart et al., 2007; Aruda et al., 2011; Pérez-Hedo et al., 2012; Lu et al., 2013; King and MacRae, 2015). Interestingly, we found that transcript abundance of two heat shock proteins, *Hsp21* and *Hsp23*, were decreased after *FABP* knockdown, indicating that *FABP* may mediate heat shock protein expression. The mechanisms by which *FABP* may affect heat shock protein transcript abundance are unknown. Perhaps *FABP* influences heat shock proteins indirectly by affecting lipid accumulation. However, FABP can also be used as a carrier to transport signaling molecules and it is possible that FABP either directly or indirectly affects heat shock proteins and other aspects of stress hardiness through this signaling (Weisiger, 2002; Furuhashi and Hotamisligil, 2008).

## Conclusion

Our proteomic screen of a time point early in the preparatory period for adult reproductive diapause in the cabbage beetle *C. bowringi* revealed 297 proteins that were differentially expressed between diapause-destined and non-diapause females. Most of these proteins fell into categories that are often associated with diapause in other studies including metabolism and fat storage, increased stress hardiness, and neuronal development. However, our study is unique and is a particularly useful contribution for two reasons. We have shown that one of the proteins up-regulated in the heads of diapause-destined females, FABP, is critical for fat accumulation during the diapause preparatory period. It is not surprising that knocking down FABP by RNAi would decrease the lipid stores that are characteristic of the diapause preparatory period in *C. bowringi* given FABP's expected role of fatty acid binding and transport along with other potential roles in lipid signaling in the insect brain through PPARs. What is surprising, and to our knowledge unique to our study, is the fact that RNAi knockdown of FABP also affected the transcript abundance of two heat shock proteins that have been associated with increased stress hardiness during diapause in other insect species. This novel finding that the increased nutrient storage and increased stress hardiness that are characteristic of diapause in so many insects may be linked through FABP certainly requires further attention, both from our group working on *C. bowringi* and other practitioners working in additional insect diapause systems.

## Author contributions

QT, WL, DH, and XW conceived and designed the experiments; QT performed the experiments; QT, WL, and XW analyzed the data; QT, WL, DH, and XW wrote the paper. WL, FZ, CL, and XW contributed materials and analytic tools. All authors read and approved the final manuscript.

### Conflict of interest statement

The authors declare that the research was conducted in the absence of any commercial or financial relationships that could be construed as a potential conflict of interest.
