# Supplementary material for: Describing the Diapause-Preparatory Proteome of the Beetle Colaphellus bowringi and Identifying Candidates Affecting Lipid Accumulation Using Isobaric Tags for Mass Spectrometry-Based Proteome Quantification (iTRAQ)
Source: Front Physiol. 2017 Apr 26;8:251. doi: 10.3389/fphys.2017.00251 (PMC5405119; doi:10.3389/fphys.2017.00251)
Supplement: Supplementary file 2 [file DataSheet2.pdf]

TiTle: Using iTRAQ to detail the diapause-preparatory proteome of the beetle *Colaphellus bowringi* and identify candidates affecting lipid accumulation

Qian-Qian Tan[1], Wen Liu[1], Fen Zhu[1], Chao-Liang Lei[1], Daniel A. Hahn[2,]*, and Xiao-Ping Wang[1,]*

1. College of Plant Science and Technology, Huazhong Agricultural University, Wuhan 430070, P. R. China. 2. Department of Entomology and Nematology, University of Florida, Gainesville, Florida, 32611, U.S.A.

*Corresponding author

Supporting Information

Table S-1. Primer sequences used for qRT-PCR and RNAi.

Table S-2. Proteins identified in the head of female adult *Colaphellus bowringi* using iTRAQ & LC − MS/MS. (Excel)

Table S-3. Differentially expressed proteins in the head of female adult *Colaphellus bowringi*. A and B refer to photoperiod of L:D 16:8 and L:D 12:12, respectively. Treatment DD was the control, and treatment NDD was the treatment group. (Excel)

Figure S-1. Functional classification of all proteins detected in the head of adult female *Colaphellus bowringi.*

Table S-1 and Figure S-1 in this file.

Table S-1. Primer sequences used for qRT-PCR and RNAi.

Primer name	(5'→3')nucleotide sequence
RPL19-qF	GTAATGCGATGCGGCAAGAA
RPL19-qR	GAGTGCACCGCTACAGGTTT
Fatty acid-binding protein-qF	TCGCGAATCATCAGAGAGCTG
Fatty acid-binding protein-qR	TTCCGGCTTCTTCAGAGTACC
Alcohol dehydrogenase-qF	GGAGGAGCTAGTGGAATCGG
Alcohol dehydrogenase-qR	CACTCTCTGGCCGTATTGCT
U6 snRNA-associated Sm-like protein LSm3-qF	CGATAGGGAAATTCGGGGCA
U6 snRNA-associated Sm-like protein LSm3-qR	TGTTCGCATTGGTGGTGAAAC
Protein phosphatase 1, catalytic subunit-qF	GCACCGAACTATTGTGGGGA
Protein phosphatase 1, catalytic subunit-qR	TGGATTTCTCTGCGGAGTGG
Cytoglobin-1-like-qF	CGCAGGATACCGGGATTCAA
Cytoglobin-1-like-qR	TTTCCGTGTATCGATCGCCC
Tricarboxylate transport protein-qF	TCGTTAAGTCATGCGACCAGA
Tricarboxylate transport protein-qR	CCCTACGACGTTACGAAATTGT
Dolichyl-diphosphooligosaccharide-qF	CACCTCGTGGAGCTCCTT
Dolichyl-diphosphooligosaccharide-qR	TTAGACGCACCATCACACCC
Protein disulfide isomerase-qF	TTTATGAGCCAAGAAGAAAATGTGA
Protein disulfide isomerase-qR	CTGACAGAATCTGCACCAAGG
GFP-T7F	GCGTAATACGACTCACTATAGGTGGTCCCAATTCTCGTGGAAC
GFP-T7R	GCGTAATACGACTCACTATAGGCTTGAAGTTGACCTTGATGCC
FABP-T7F	GCGTAATACGACTCACTATAGGCAGGGTACTCTGAAGAAGCCG
FABP-T7R	GCGTAATACGACTCACTATAGGATAGCCTCTTTGCTTCCAGACC

Figure S-1

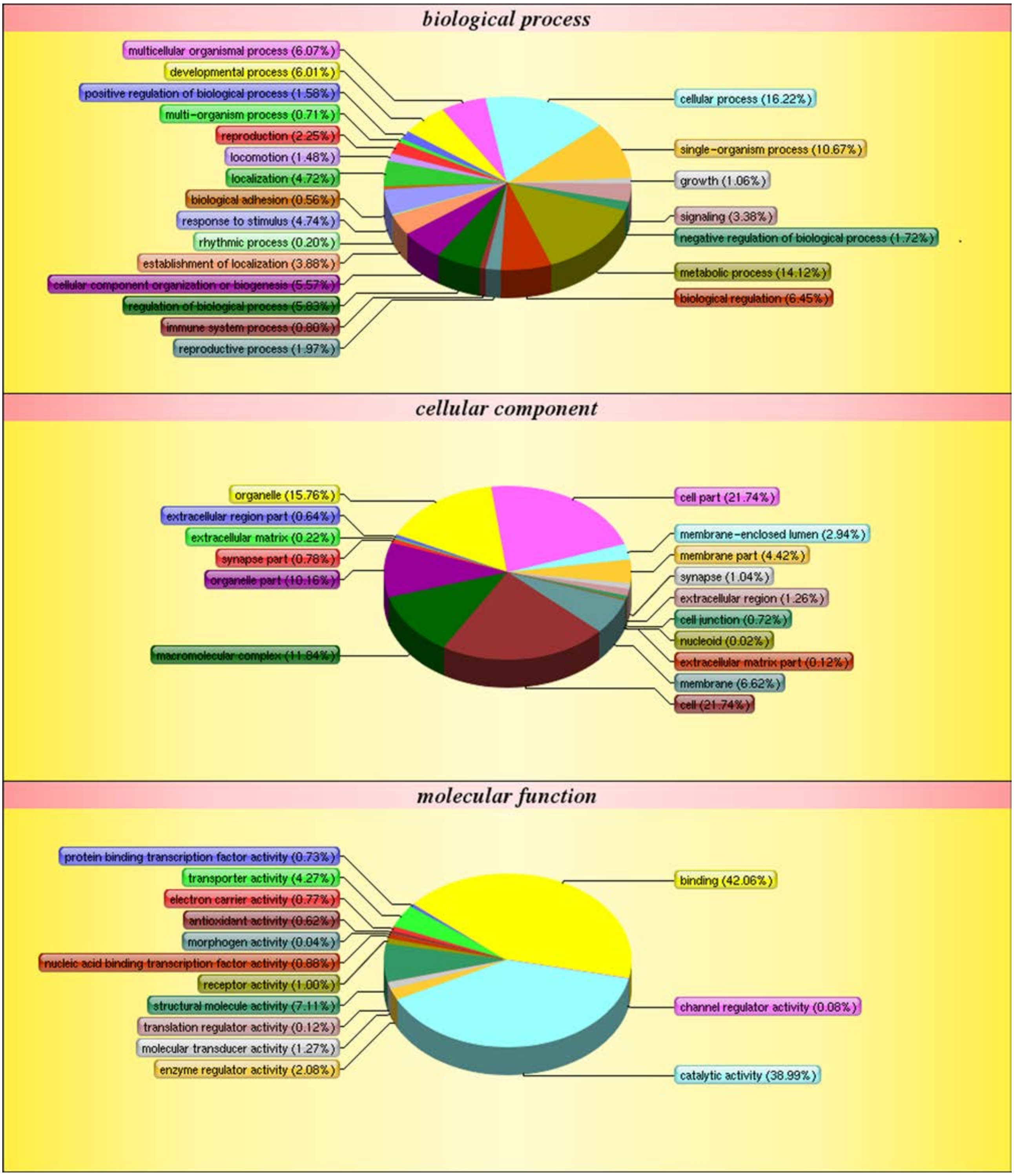

Figure S-1. Functional classification of all proteins detected in the head of adult female *Colaphellus bowringi.*
